# The Standard Bearer

**Published:** 1913-11

**Authors:** 


### The Standard Bearer.

Who was it first took fire at the cruel exploitation of children in industry; that cried aloud to halt greed's slaughter of the innocents?

Woman.

Who was it organized the fight on child labor, gathered the evidence, prodded the lazy legislators, camped on the trail of the factory inspectors, wrote letters to the newspapers and collected the sinews of war?

Woman.

Who, unwearied by discouragement, by ridicule, by opposition, has kept up the unending battle on the abuse of alcohol?

Woman.

Who put heart and soul and punch into the fight on the social evil, and especially into the fight to halt greed's dooming of young girls to commercialized lives of sin?

Again woman.

Whose is the conscience and the moral courage which is slowly putting to shame the entire structure of business inhumanity so that there may take its place a relationship more nearly akin to brotherhood? Who are the moral militants in the struggle for social justice?

Woman, always woman.

There are sensitive, morally courageous men, who dare greatly to do service for humanity. But every one is the son of a woman; every one got his finest training from a woman; every one would be powedless but for the smypathy and support of women.—*Austin Daily Tribune.*

